# Locked‐in syndrome: A qualitative study of a life story

**DOI:** 10.1002/brb3.3495

**Published:** 2024-08-06

**Authors:** Mona Lisa Hordila, Cristina García‐Bravo, Domingo Palacios‐Ceña, Jorge Pérez‐Corrales

**Affiliations:** ^1^ Activa Center Ferrol A Coruña Spain; ^2^ Research Group of Humanities and Qualitative Research in Health Science, Department of Physical Therapy, Occupational Therapy, Physical Medicine and Rehabilitation Universidad Rey Juan Carlos Alcorcon Spain

**Keywords:** life story, locked‐in syndrome, narrative, qualitative research, qualitative study

## Abstract

**Introduction:**

Locked‐in syndrome (LIS) is characterized by tetraplegia, anarthria, paralysis of cranial nerves, and facial musculature, with the preservation of consciousness and cognitive abilities, as well as vertical eye movements and eyelid movements, hearing, and breathing. Three types of LIS are distinguished: classic, incomplete, and total. The aim of the present study was to describe the life history of a person with LIS, as well as the wife's experience and perspective of this life history.

**Methods:**

A qualitative life history study was conducted with two participants: a 54‐year‐old man diagnosed with LIS and his 50‐year‐old wife. Data were collected through interviews and autobiographical documents submitted by the participants and analyzed following Braun and Clarke's method of inductive thematic analysis.

**Results:**

Five main themes were identified: (1) how to understand and overcome the new situation; (2) the process of care and rehabilitation; (3) communication; (4) writing as a way of helping oneself and others; and (5) personal autonomy and social participation.

**Conclusion:**

The participants valued the support of their friends and family in the acceptance stage of the new situation, giving special importance to the communication skills and medical attention received after diagnosis.

## Graphical abstract

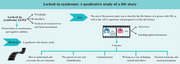

## 1 INTRODUCTION

Locked‐in syndrome (LIS) is characterized by the presence of tetraplegia, anarthria, paralysis of lower cranial nerves, and facial musculature, with the preservation of consciousness and cognitive abilities (Nikic et al., 2013; Papadopoulou et al., 2019; Plum & Posner, 1996; Schjolberg & Sunnerhagen, 2012;) as well as vertical eye movements and eyelid movements, hearing and breathing (Vidal, 2018). Due to the total immobility of those affected, it is necessary to perform an electroencephalogram (EEG) to check the presence of consciousness (Svernling et al., 2019). Three types of LIS are distinguished according to the degree of mobility present: classic, when eye movements are present and consciousness is maintained; incomplete, when residual voluntary movements other than eye movements are present and consciousness is maintained; and total, when there is total immobility, including the absence of eye movements, and consciousness is maintained (Bauer et al., 1979). However, due to the intricate nature of the symptoms defining LIS, accurate diagnosis might be challenging, necessitating a differential diagnosis to distinguish it from other conditions like unresponsive wakefulness syndrome, minimally conscious state, and akinetic mutism (Schnetzer et al., 2023). Verification of consciousness often requires tests such as an EEG (Bauer et al., 1979; Svernling et al., 2019), coupled with imaging assessments such as magnetic resonance imaging (Schnetzer et al., 2023). The main causes of LIS are cerebrovascular accident, traumatic brain injury, degenerative diseases, such as amyotrophic lateral sclerosis (Aust et al., 2022), and the presence of tumors (Nikic et al., 2013; Schjolberg & Sunnerhagen, 2012; Vidal, 2018), causing injury to the basilar artery area (Raibagkar et al., 2017; Svernling et al., 2019). The prevalence of LIS is not known exactly but it is between 0.17/10,000 and <1/1000,000 (Farr et al., 2021).

According to the International Classification of Functioning (ICF), Disability, and Health (World Health Organization [WHO], 2001), there exists an intricate interplay between health conditions and environmental factors that significantly affect individuals' participation and performance. It is important to underscore the disease's impact on the quality of life for individuals with LIS and their spouses or closed relatives. The limitations resulting from LIS directly impact their daily life, potentially restricting activities and participation within their environment.

Different studies analyze the clinical, functional, and quality‐of‐life (QoL) implications of people with LIS (Haig et al., 1987; Nikic et al., 2013; Papadopoulou et al., 2019; Patterson & Grabois, 1986; Pistoia et al., 2016; Rousseau et al., 2015; Schjolberg & Sunnerhagen, 2012; Svernling et al., 2019; Vidal, 2018). However, there is scant literature reflecting the experience of people with LIS from their point of view and that of their relatives.

The aim of the present study is to describe the life history of a person with LIS, as well as the wife's experience and perspective of this life story.

## 2 METHODS

### 2.1 Inclusion criteria

A case study was conducted (Anguera Argilaga, 1986) in which one main participant, the study subject, and his wife were included. The inclusion criteria for the patient were as follows: being over 18 years old; having a diagnosis of Incomplete LIS or Classic LIS, having written communication skills through eye movements on eye tracking devices, or through body movements that allow you to operate a keyboard or joystick to type on a computer, thus being able to answer semi‐structured interviews. The inclusion criterion for the family member was as follows: being a direct family member of the affected person.

### 2.2 Ethical considerations

Before starting the study, the participants gave their informed consent. Both participants were provided with the informed consent document via email a week prior to the interviews. They had ample time to review, comprehend, and address any queries before formally signing and consenting to participate in the study. On the day of the semi‐structured oral interview with the main participant's wife, physical copies of the documents were presented for both participants to reexamine and sign before commencing the interview. The main participant was able to sign using a pen to provide his signature. The study was accepted by the ethical committee of Universidad Rey Juan Carlos (code: 1604202110821).

### 2.3 Procedure

A qualitative life history study was conducted (Berenguera Ossó et al., 2014) in which the experience of the subject with LIS before, during, and after diagnosis is described, as well as the experience and perspective of his wife. Purposeful sampling was carried out (Creswell & Poth, 2018).

Qualitative research is used in response to a need to listen to silenced voices, empowering people to tell their stories and understand the contexts in which they address their problems (Creswell & Poth, 2018). Among the different forms of qualitative study of life histories, the single‐story technique consisting of collection and analysis of the person's experience has been chosen, complemented with the technique of crossed stories (Berenguera Ossó et al., 2014) in order to explain the same life story from different perspectives. The life history technique in qualitative research offers unique depth by exploring the entire life of an individual, allowing a detailed and contextualized understanding of their experiences over time. This methodological approach stands out for its ability to capture the evolution of events, values, and decisions, generating a holistic perspective (Berenguera Ossó et al., 2014). The study has been conducted in accordance with previous international studies (Fernández‐Moya et al., 2022; Gillard et al., 2022; Harel et al., 2022; Kivnick et al., 2020). Gillard et al. (2022), Harel et al. (2022), and Kivnick et al. (2020) conducted single case studies, employing solely semi‐structured life‐history interviews with a single participant, subsequently transcribing and analyzing them using thematic analysis. Our study's strength lies in conducting not just two semi‐structured interviews with the participant with LIS but also expanding our data through interviews with the participant's wife (employing the technique of crossed stories) (Berenguera Ossó et al., 2014), analyzing information from two published books by the participants, a radio interview, a documentary, and researchers' field notes. This comprehensive approach enhances the richness and depth of our results (Carpenter & Suto, 2008), aligning with a widely used quality criterion in qualitative studies: triangulation across multiple data sources (Lincoln & Guba, 1985). Fernández‐Moya et al. (2022) also employed triangulation in their single case study of a participant with a spinal cord injury, gathering data through an in‐depth interview and personal writing from the participant. Nonetheless, our study incorporates a more extensive array of data sources.

### 2.4 Data collection

Data collection for this research study was carried out through in‐depth semi‐structured interviews, field notes made by the researcher, and personal documents provided by the participants.

As the subject with LIS has lost the ability to speak, he answered the questions in writing. With the ability to move the first finger of his right hand, the subject can operate a joystick to write on a computer. As his wife does not have communication problems, his interview was conducted in a conventional way: He was interviewed in person orally, and, with his wife's permission, the interview was recorded, complementing this information with the researcher's field notes. The interview lasted 78 min. The semi‐structured interviews questions have been included in Supplementary Material 1.

To give greater consistency to the research and expand the knowledge about the experiences of the participants in the study, they were required to voluntarily provide complementary documents that reflected their experience, thus adding to the information provided in the in‐depth interviews (Carpenter & Suto, 2008). The subject with LIS submitted for analysis two books that he had written and an interview, conducted after having been diagnosed with LIS on a radio program that lasted 29 min and 43 s. This interview was carried out through a computer program which orally reproduced what the participant wrote on his computer. To this, a documentary was added about the lives of both participants that they recorded in 2004.

### 2.5 Data analysis

**FIGURE 1:**
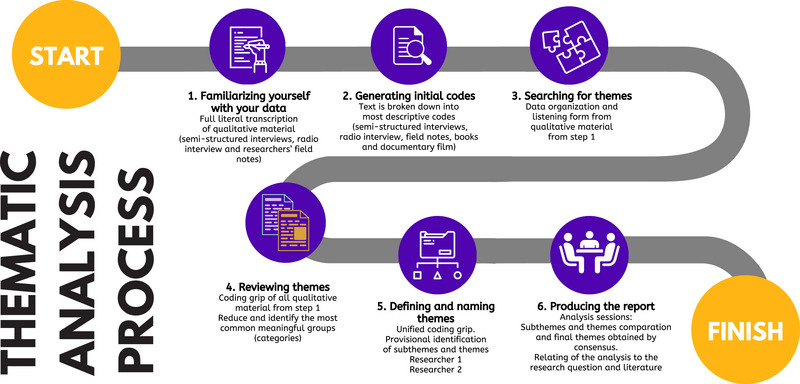
Thematic analysis process (Braun & Clarke, 2006).

After conducting the interview with the wife of the subject with LIS, the recording was transcribed verbatim and completed with the field notes taken by the researcher. In addition, the subject with LIS provided the researcher with an audiotape of an interview he gave to the radio. This interview was transcribed to be analyzed, proceeding in the same way as with the documentary that the wife delivered. To analyze the data obtained through the two interviews and the answers to the questions sent to the subject with LIS, which are received in written form, as well as the information from the two books and the documentary, the method of inductive thematic analysis of Braun and Clarke (Braun & Clarke, 2006) was followed. Inductive thematic analysis guides a coding process that does not try to fit the data into a preexisting theoretical framework or through preestablished categories, so the different codes emerge from the narrative of the participants (Braun & Clarke, 2006). Researchers fragment the entire data into smaller units of meaning, which are coded, and named according to the content they represent, and later grouped into common categories (Moser & Korstjens, 2018). In this way, different thematic levels were created (codes that gave rise to categories, categories to subthemes, and subthemes to main themes) that were grouped, named, and defined through consensus between researchers established in research meetings (Figure 1). No software was used for data analysis.

In carrying out this study, the recommendations established by the Consolidated Criteria for Reporting Qualitative Research (Tong et al., 2007) (Supplementary Material 1) and the Standards for Reporting Qualitative Research (O'Brien et al., 2014) have been followed.

## 3 RESULTS

Two participants have been included in the study, a lead participant. Both reside in a Spanish city with a population close to 300,000 inhabitants and have been married since 1999. The man is 54 years old, and the woman is 50 years old. The main participant was diagnosed with Incomplete LIS as a consequence of a stroke in 1999. The woman does not have any significant pathology related to this research. At the time of the interview, the subject with LIS lived in a residence for people with disabilities. The wife lived at home is her husband's primary caregiver and legally has conservatorship for him. Additionally, she maintains an active work situation as an administrative staff member in a company, the same position she held at the time when her husband suffered the stroke that led to LIS. Before being diagnosed with LIS, the main participant in this study worked as an accountant. Both participants have vocational training backgrounds, and their shared hobbies include watching sports such as rugby and handball, as well as going to the cinema.

The signs of the lead subject's disease meet some of the characteristics described above for Incomplete LIS (Bauer et al., 1979): Anarthria and generalized immobility can make slight body movements and have preserved cognitive abilities. He is able to communicate through an eye‐blinking system. With this system, he can use the computer to communicate as well as to surf the Internet and write, as he is not able to write with his hand using a pen other than to sign.

**FIGURE 2:**
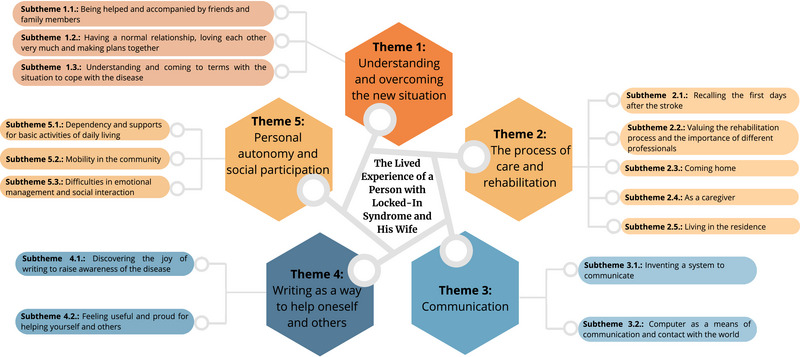
The distribution of themes and subthemes on the experience of a person with locked‐in syndrome (LIS) and that of his wife.

The findings of this research have been organized into five main themes in which the experience of a person with LIS (P.1) and that of his wife (PA.1) is described. The distributions of themes and subthemes are shown in Figure 2 and categories in Supplementary Material 2.

The participants describe their most relevant experiences as feeling supported by friends and family as well as the healthcare received in the hospitalization stage and in the later stages when the wife assumed the role of primary caregiver. In addition, both participants highlight the writing of two books as a method of self‐help and acceptance of the situation, while also giving importance to the limitations that the person with LIS has for the development of activities of daily living (ADL).

### 3.1 Theme 1: Understanding and overcoming the new situation

Both participants describe the experience of feeling supported, placing great importance on social support during the time they were in the hospital and the help that their relatives gave them when the participant with LIS lived at home.

That is very important when you have a strong problem, which is difficult to overcome, that there are people supporting you and helping you. (PA.1, semi‐structured interview)

The participant with LIS was diagnosed 3 months after getting married, something that interrupted the plans they had as newlyweds, although currently both express having a normal relationship, despite the opinions of others. From the participants' perspective, having a normal relationship means being a married couple that stays together despite not living in the same house.

I think people think our relationship is not a normal relationship. I don't think they even see us as a couple. We don't live together. (PA.1, documentary film)

Therefore, as both describe in the interviews, the family members did not tell him the truth until months later when he himself deduced his situation, suffering a very hard subsequent period. The wife explains that the main reason why they did not tell her the truth was so that her husband could try to recover as much as possible and not give up because he thought that his illness would not allow him to regain any movement.

I was the one who found out I couldn't express my thoughts and it really was a big stick that took me quite a while to recover from. (P.1, radio interview)

### 3.2 Theme 2: The process of care and rehabilitation

During the 9 months that the main participant was admitted to the hospital, the wife reports that she saw the small movements that he made and was sure that they were voluntary, but when she told the doctor he did not believe her, causing feelings of frustration. Initially, physicians conveyed to them that the primary participant would only retain the ability to move their eyes, suggesting a Classic LIS. However, the wife persisted in showcasing that the main participant could perform additional small voluntary movements, leading to a revised diagnosis from Classic LIS to an Incomplete LIS.

They told us that he would only be able to move his eyes and that he would never be able to move any part of his body. That he was perfectly fine, that he was perfectly fine but that the only thing he would be able to move were his eyes. (PA.1, semi‐structured interview)

The one who communicated to the doctor that I thought I had seen a glimmer of voluntary movement in me was P. At that time I tried, but I never could, and the feeling is one of total frustration. (P.1, semi‐structured interview)

I noticed small movements, and I mentioned it to the doctor, but he disagreed, attributing them to spasms. Despite my insistence and pointing it out, the doctor insisted that my observation was incorrect and that the patient didn't respond when asked to move. (PA.1, semi‐structured interview)

Both participants report that the rehabilitation that the participant with LIS received was passive during the time they were admitted to the hospital and a lack of medical follow‐up after discharge. Both agree that he should have received “more thorough” or active rehabilitation. The passive rehabilitation consisted of mobilizations that the therapists performed on the person with LIS passively, without asking for voluntary movement from the participant.

The doctor concluded that he wouldn't move beyond his eyes and that he was unlikely to improve. The rehabilitation provided mainly involved repositioning him physically, but it wasn't extensive (PA.1, semi‐structured interview)

As the doctor decided that he was not moving and was never going to move, he was not going to move more than his eyes, because the rehabilitation they gave him was that they moved him, not that … but it was not much (PA.1, semi‐structured interview)

If there is something that I reproach him to a greater extent is that for much of the time I was under his (the doctor's) tutelage, the rehabilitation that was done to me was passive. (P.1, book 1)

Currently, the main participant receives psychological care, which he considers important to understand his situation and continue living despite the disease. He admits to not understanding why he was not offered this during his hospital stay.

I think a case like mine, psychological treatment was important, but I don't know why I only had one visit. (P.1, semi‐structured interview)

During the period that the main participant lived in the family home, his wife took on the role of primary caregiver. She describes an apprenticeship period in which she needed the help of her in‐laws. Being a caregiver for a totally dependent person and maintaining an active work situation meant a physical and social life deterioration as the wife of someone with LIS, as she describes in the interviews.

And either I was already a zombie, all day working and such and not sleeping at night (…) Well, there is no body that can stand it … My social life … I didn't leave the house. We didn't leave the house. (PA.1, semi‐structured interview)

As a person with LIS, being aware of his own care needs and the effort that his family was making to meet them was the reason that he describes as being responsible for his voluntary decision to enter a residence.

I have to say that the decision to enter a residence was mine. because I already told you that I felt a burden to P. and to my parents. (P.1, semi‐structured interview)

### 3.3 Theme 3: Communication

Both participants describe how, at first, the main subject with LIS communicated through the blinking of his eyes, answering closed‐ended questions with a yes or no. His wife explains the difficulty of communicating in this way. Both recognize that the ability to communicate is the most important and fundamental thing, so they needed to invent a communication system and, when they succeeded, the main participant describes it as the best day of his life.

I can communicate with the outside world with a flick of my finger. (P.1, book 2)

That communication is fundamental in this type of patient and in anyone, is the most important thing. (PA.1, semi‐structured interview)

They created a system through the blinking of the eyes and a template in which the letters of the alphabet are divided into four rows.

Yes, everyone communicates with me with that letter sheet (..) I blink in the row that is and they tell me the letters of the alphabet in that row. (P.1, semi‐structured interview)

In addition to the possibility of communicating through the letter sheet and the blinking of his eyes, the participant with LIS uses the computer to make himself understood. He describes the computer and the programs that he uses (both the joystick and the eye control) as a tool of great importance in his life, thanks to which he is able to communicate with many people he otherwise could not, thus having greater independence.

In general, I am happy because the computer allows me to look less shabby than I really am. (P.1, semi‐structured interview)

### 3.4 Theme 4: Writing as a way to help oneself and others

After being diagnosed with LIS, the main participant wrote two autobiographical books. Regarding this, he admits that before suffering the stroke, he had not thought about writing something of his own and the two books have no literary intention other than to publicize the disease. Writing makes him feel useful, as he believes that it provides practical tools on assistive products and communication systems.

I think that without intending it is emotional, although many things are also practical, a good chair, a good rehabilitation, access to the computer, etc. (P.1, semi‐structured interview)

He wrote the book with the intention of aiding others, and the best part is that he succeeded, which fills him with immense pride. (PA.1, semi‐structured interview)

In addition to helping other people, something the main participant admits to being proud of, books have also been a means of accepting their situation.

As you write the book, of course, it helps you accept the situation. (P.1, radio interview)

### 3.5 Theme 5: Personal autonomy and social participation

The participants highlight the affectation of basic ADLs, giving special importance to activities related to eating and drinking. They describe an evolution in the feeding capacity of the main participant with LIS. Currently, the main means by which he feeds is through a gastrointestinal tube, although he is able to ingest some foods that do not require chewing, something he values very much. He reports that he can drink almost everything if it is given through a syringe.

If someone had told me I was going to be able to eat and drink by mouth, I would have sworn I was crazy. (P.1, semi‐structured interview)

In addition to the difficulties that the person with LIS has for basic ADLs, the participants describe the complications they encounter when traveling around their city with the subject in a wheelchair.

I was barely advised on the purchase of my first wheelchair and that bad purchase took away a lot of possibilities for ‘the evolution of my life’ for a period of 4 years. (P.1, book 2)

It's challenging. Eventually, as with everything, you have to adapt because there are places we can't access, which is impossible. There are certain places we'll never be able to enter. (PA.1, semi‐structured interview)

As described above, the participants admit to having felt supported by their friends and family and currently have a lot of social life. However, the person with LIS describes some difficulties related to his social interaction skills. He admits to not being able to fully control his emotions, describing mood swings and uncontrolled crying.

If there is the loss of someone close to me, attendance at their funeral becomes practically impossible, because as I could see in the only emotional ceremony I went to, my cries were so intense that I noticed how the people close to me felt really uncomfortable. (P.1, book 1)

Furthermore, in relationships with others, the main participant describes situations in which the people around him either sympathize with him or do not address him directly because they think he does not understand them.

She came into my room and asked my wife (as if I was dumb or something similar and couldn't answer by nodding) if I liked motorcycling, when I think she could and should have addressed me. (P.1, book 1)

## 4 DISCUSSION

The results of the study suggest that the most important areas in the participants' lives are social support, rehabilitation, and communication skills. In addition, the person with LIS is proud to have been able to participate in expanding the knowledge about the disease by writing two books.

The results of our study show, from the participants' perspective, a relationship between being diagnosed with LIS and the domains included in the ICF (WHO, 2001). Specifically, communication, mobility, self‐care, interpersonal interactions, and community and social life are affected.

Notably, limitations are highlighted in the categories of communication and mobility within the activity domain included in the ICF (WHO, 2001). According to the results of the present study, communication is essential for the participants. The main participant uses a means of communication based on the use of letter boards and computer programs. In the semi‐structured interview carried out with the main participant, he refers to preferring to use communication through eye blinking and the letter template whenever he communicates with his wife, as, after many years of practice, it is the method with which they easily communicate. However, if he wants to have a conversation with a person who is not in his close social circle and does not know the eye blink and letter template system, he prefers to use a computerized system, as it is a faster method. These results are consistent with previous studies, such as that of Lugo et al. (2015) in which participants were able to communicate using a different code than Yes/No, such as blinking.

However, most preferred to use alphabetic codes or body movements to communicate, even though more advanced electronic means were available (Lugo et al., 2015).

Rousseau et al. (2015) concluded in his study that although participants use electronic devices to communicate, the most common form of communication in patients with LIS remains through blinking and eye movements, on boards of letters or symbols. The study by Nizzi et al. (2020) highlights the importance of communication in people with LIS as their only channel of action, after having lost the ability to express themselves.

The participants in this study consider that a more active rehabilitation would have improved the QoL of the person with LIS, and that of his wife who adopted the role of main caregiver, both suffering a deterioration of their physical and social life. These results are consistent with studies such as Lugo et al. (2017), in which the needs, QoL, and psychological state of people with LIS and their families were analyzed. Most of the participants complained of problems related to patient care and the possible solutions offered to them, encountering great difficulties in accessing information and adequate rehabilitation centers, as well as problems with medical equipment and a lack of emotional support for family members (Lugo et al., 2017). Lugo et al. (2017) referred in their article to the fact that, in more than half of LIS cases, family members put the patient's well‐being before their own. Schjolberg and Sunnerhagen (2012) conducted a study examining the rehabilitation intervention provided to four LIS patients by a multidisciplinary team, characterized by the authors as a comprehensive unit comprising a nurse, nursing assistant, physiotherapist, occupational therapist, swallowing specialist, physician, and communication team. Their findings emphasize the necessity of comprehensive rehabilitation for individuals with LIS, involving diverse professional interventions. However, Schjolberg and Sunnerhagen (2012) noted that due to the condition's rarity, there might be instances where a proper evaluation and treatment are not administered. In the same vein, Halan et al. (2021) underlined the importance of multidisciplinary intervention in the rehabilitation process of patients with LIS, as well as the importance that their families give to access to quality information through medical services.

As mentioned earlier, the primary participant in this study initially received a diagnosis of Classic LIS. However, this diagnosis was later revised to Incomplete LIS, possibly due to the absence of standardized tests for confirming LIS diagnoses. Patients in such cases are frequently heavily sedated and have brief interactions with healthcare professionals, hindering a thorough clinical evaluation. It can take up to 2 months to conclusively confirm the LIS diagnosis, impacting the functional recovery process for these patients (Surdyke et al., 2017). Although some studies advocate for comprehensive and active rehabilitation, there is currently insufficient evidence regarding the effectiveness of physical exercise in the recovery of LIS patients. To formulate a therapeutic plan, physicians must integrate insights from their professional experience, current research, and evidence as well as consider the individual needs of the patient (Law et al., 2018). Thus, to develop safe and precise exercise programs, it is crucial to understand the specificities of patients, their families, and caregivers through effective doctor–patient–family communication. As highlighted by Arteaga Mendieta and Zea Vera (2014), communication serves as a fundamental tool through which doctors convey information about the diagnosis and prognosis of a pathology. Respecting the patient's freedom of opinion and autonomy, and avoiding a paternalistic approach where the doctor's opinion takes precedence, is crucial in fostering positive doctor–patient–family relationships (Fernándes et al., 2022). This, in turn, promotes understanding and adherence to the treatment of stroke patients (Cheloudaki & Alexopoulos, 2019). Fernándes et al. (2022) further emphasized that communication barriers, such as a lack of empathy, failure to anticipate the prognosis, estimate life expectancy, and provide guidance and follow‐up, can adversely affect doctor–patient–family relationships.

Following the structure of the ICF, we can relate the findings of this study to significant restrictions in participation. Regarding self‐care, it is reflected that the capacity that the main participant of this study has in the development of ADLs is very poor. Taking into account the limitations that affect the functionality of people with LIS in ADLs, Schjolberg and Sunnerhagen (2012) analyzed the necessary care of subjects with LIS, concluding that patients with this diagnosis require a gastric tube for correct and safe feeding. In contrast, Maiser et al. (2016) mentioned that, eventually, patients end up removing the gastric tube.

Regarding interpersonal interactions and community and social life, in the first theme of our study's results, both participants emphasize the importance of the social support they have received since the main participant suffered the stroke that caused the LIS. The results obtained in this study are consistent with the results of the study by Lulé et al. (2009) in which it is concluded that one of the most important factors that helps people cope with a disease like LIS is social support. It is crucial to consider this aspect as highlighted by León‐Carrión et al. (2002) in their study, indicating that the majority of individuals with LIS find enjoyment in social outings and gatherings, albeit requiring assistance due to their dependency. Through the aid of friends and family in organizing social activities, those with LIS can lead fulfilling lives and actively engage in society. The primary participant in this study faced the onset of LIS 3 months post‐marriage, and as indicated in the analyzed interviews, the circumstances following the LIS diagnosis were challenging, yet they persevered, remaining united in marriage. This aligns with the findings of León‐Carrión et al. (2002), stating that only 9.1% of couples separate after one spouse is diagnosed with LIS. Consequently, the study's results underscore a significant correlation between the reported QoL of the individual with LIS and their spouse, emphasizing the pivotal role of social support during hospitalization and subsequent stages. In‐line with these results, we find articles such as that of Kuzma‐Kozakiewicz et al. (2019) who analyzed the QoL and affective state of people with LIS, proposing that they can adapt emotionally to the new situation if they have strong social support. Along the same lines, Rousseau et al. (2013) highlighted the importance of caregiver support, psychological support, and the social environment as factors that impact the quality of life of people with LIS. Moreover, the bibliography contains intriguing insights like the disability paradox articulated by Albrecht and Devlieger (1999). This concept elucidates that numerous individuals grappling with severe disabilities, including those with LIS, perceive their quality of life as excellent or good. Conversely, some doctors and health professionals hold the belief that individuals facing limitations in their daily activities endure an unsatisfactory quality of life. Despite this paradox, the psychological issues that can occur in patients with LIS remain relevant. Indeed, when assessing the quality of life among individuals with LIS, an important consideration involves the prevalence of poststroke depression, impacting both patients and their families (Lugo et al., 2017; Shewangizaw et al., 2023). The primary participant in this study reported experiencing a phase of depression post‐LIS diagnosis, highlighting the absence of psychological support. Various studies have investigated depression in stroke survivors (Shewangizaw et al., 2023) and LIS patients (Halan et al., 2021). The systematic review by Halan et al. (2021) showed that depression is a long‐term complication in patients with LIS. Consequently, recognizing these emotional challenges is crucial, as they can significantly impact both patient rehabilitation and the well‐being of their families (Halan et al., 2021; Lugo et al., 2017).

The main participant presents the narration of personal experiences through autobiographical books as a valuable tool to accept and overcome their situation. Vidal (2018) conducted a study in which he analyses different autobiographical texts of people with LIS. He considers these narratives as *a tool to explore the social, cultural, symbolic, relational and psychological dimensions of the disease, and to improve the empathic understanding of the experience of patients and caregivers* (Vidal, 2018, p. 51). Ruini and Mortara (2022) reviewed “writing therapy” as a psychotherapy technique to understand and overcome traumatic experiences. In one of the books written by the main participant and analyzed in this study, situations are described in which the participant with LIS has not been able to control his emotions, experiencing episodes of emotional lability or uncontrolled crying. These episodes are frequently experienced by people with LIS, such as the cases reflected in the article published by Smith and Delargy (2005), in which 39 of the participants with LIS acknowledged that they cried or laughed more easily than before. Likewise, Sacco et al. (2008) described similar situations of emotional lability or uncontrolled laughing and crying as inappropriate emotional episodes in a given uncontrolled situation that do not correspond to the feelings of the person experiencing them.

One of the complaints of the main participant is that some people do not realize that he understands the questions that may be asked and there is no need for him to ask his caregiver. In their article, Nizzi et al. (2020) clarified that patients with LIS may feel as if they are treated as objects, so it is important to adopt an appropriate attitude when communicating and to address them directly instead of talking to their caregiver.

## 5 LIMITATIONS

This work has a number of limitations. Due to the low prevalence and characteristics of people with LIS, it was not possible to collect data from a larger number of participants. Likewise, the technique used to conduct the interview with the main participant has not allowed everything to be evaluated in depth for some aspects. However, a number of documents have been included to complement the information provided by the participant.

### 5.1 Future directions

This work could serve as a starting point for future qualitative research on the experiences of people with LIS as well as to address the relationship between the experiences of people with LIS and the care offered in health systems, as well as the care of their primary caregivers.

## 6 CONCLUSIONS

This study analyzes how a person with Incomplete LIS and his wife have experienced the different stages as the main participant of the study was diagnosed with LIS. Participants describe their life and relationship as “normal,” maintaining a proper social life, traveling, and making plans with friends and family. The main participant was the one who became aware of his diagnosis, describing those moments as very hard as well as falling into a depression.

Among the most notable themes, we find communication, in which both participants describe as the most fundamental aspect, and the rehabilitation received mainly in the first months after suffering the stroke that caused the LIS. Both participants rate the rehabilitation received as poor and describe a lack of active rehabilitation and medical assistance during the time they remained in the hospital as well as after receiving hospital discharge.

Despite suffering from a serious motor impairment that has caused him total dependence in the basic and instrumental activities of daily life, the main participant has written two autobiographical books, which, as he explains, have helped him accept and overcome the new situation.

## AUTHOR CONTRIBUTIONS

**Mona Lisa Hordila**: Conceptualization; methodology; formal analysis; investigation; writing—original draft; writing—review and editing; visualization. **Cristina García‐Bravo**: Methodology; formal analysis; data curation; writing—review and editing; visualization. **Domingo Palacios‐Ceña**: Methodology; investigation; data curation; writing—review and editing; supervision. **Jorge Pérez‐Corrales**: Conceptualization; methodology; formal analysis; investigation; data curation; writing—original draft; writing—review and editing; visualization; supervision; project administration.

## CONFLICT OF INTEREST STATEMENT

The authors have no conflicts of interest.

## FUNDING INFORMATION

This research has not received specific grants from public sector agencies, the commercial sector, or nonprofit entities.

### PEER REVIEW

The peer review history for this article is available at https://publons.com/publon/10.1002/brb3.3495.

## Supporting information

Supplementary Material 1

Supplementary Material 2

## Data Availability

The data that support the findings of this study are available on request from the corresponding author. The data are not publicly available due to privacy or ethical restrictions.
