# Supplementary material for: Locked‐in syndrome: A qualitative study of a life story
Source: Brain Behav. 2024 Aug 6;14(8):e3495. doi: 10.1002/brb3.3495 (PMC11302814; doi:10.1002/brb3.3495)
Supplement: Supplementary file 1 — Supplementary Material 1 [file BRB3-14-e3495-s001.docx]

**SUPPLEMENTARY MATERIAL 1**

**Questions from the first semi-structured interview conducted with Participant 1:**

1. What was your childhood like? What stands out in your memory? Did you have any particular hobbies?
2. Can you describe your youth? What stands out in your memory? Any specific hobbies or interests?
3. How did you enter the workforce? At the time of the stroke, you were working in a construction company. What roles did you hold? Did you have previous job experiences?
4. When and how did you meet your wife? Can you talk about your relationship before the stroke?
5. What was your typical routine in the weeks preceding the stroke? Can you describe your activities on a workday and a weekend?
6. What activities were significant for you before the stroke? (self-care, household, social, leisure, etc.) Why were they important?
7. Currently, you communicate through writing. Did you engage in this activity before the stroke? If so, how did it start and why?
8. During the stroke, you were alone at home. Can you describe your experience during that time?
9. What were your feelings when you woke up and couldn't communicate?
10. You previously mentioned that your family didn't immediately inform you of your diagnosis. How did you find out? Your thoughts upon learning? Did you understand their reasons? Would you have preferred to know from the start?
11. Upon admission, you were in the hospital for nine months. Can you share your experience? Types of treatments received and their effectiveness? How did the duration of hospitalization affect your acceptance of the diagnosis? Would you have preferred to stay or go home? Your emotions upon being told you could leave? Expectations for recovery then?
12. You mentioned the crucial role of family support in accepting the diagnosis. How did they assist you?
13. What treatments have you undergone and how do you rate them?
14. Your first successful communication after the stroke—can you describe that experience? How did you adapt to the communication system using letter tables and eye-blinking with your wife's help? Why do you prefer this method over screen-based communication?
15. You mentioned maintaining some hobbies like sports. Do you still enjoy them as much? Differences in experience now? Sports activities you had to give up? Your emotions then and now? New hobbies due to your condition? Describe the emotions involved.
16. How has the inability to engage in previous activities affected relationships with friends and family? In general, how has your family and social life been impacted?
17. Describe your current daily routine. Any satisfaction or desire for changes given your circumstances?
18. Your current residence—how was the transition? Adaptation? Relations with other residents and staff? Communication methods and challenges? Why?
19. Rugby's significance to you—your emotions attending games? Writing articles on rugby—when and why? Other enjoyable activities now? Any desired activities not currently pursued?
20. You mentioned encountering architectural barriers in the city. Describe these experiences and feelings.
21. Recall any situations where you felt treated indifferently or underestimated. Details? Emotions?
22. How would you define your current life? What do you miss the most? Assessment of family and social life?
23. From the details you shared about a previous interview, you mentioned that you 'no longer count your dreams' and have refrained from making future plans. When did this change occur? Do you still hold the same perspective, or has there been a shift in this regard? Do you currently have any expectations or plans for the future?"

**Questions from the second semi-structured interview conducted with Participant 1:**

1. You mentioned that you and your wife enjoy going to bars as a hobby. How has this activity changed since the Cerebrovascular Accident (CVA)? What has been your experience over the years?
2. You expressed a desire to travel after getting married. Did you manage to take any trips together before or after the stroke? If so, how did you experience them? Any challenges related to your condition? How has traveling changed post-stroke?
3. Another activity you and your wife shared was going to the movies. How did this activity change? What has been your experience over the years?
4. You mentioned feeling like a 'simple number' to your employer despite your efforts. Can you elaborate on this?
5. You used to enjoy leaving Valladolid on weekends before the stroke. Where did you like to go? Is this something you still do?
6. You mentioned maintaining your social relationships. Have they changed in any way due to the stroke? How?
7. You spoke about a trip to the US and said that given what followed, you have no regrets. Are there things you wished you had done?
8. You found limited information about Captivity Syndrome after your diagnosis. Can you recall what those cases mentioned? How did it make you feel, and what information were you seeking?
9. You mentioned receiving correspondence from individuals with Captivity Syndrome. How do you feel your books have helped them, practically or emotionally? Can you share examples? How do these interactions make you feel? What did achieving your goal of raising awareness mean to you? When and why did you decide to create the documentary Verbos? Can you share significant anecdotes for a potential future book?
10. Can you recall the moment when you sought help from your neighbors after the first stroke? Can you provide more details?
11. You mentioned experiencing 'Yupi's worlds' when first waking up and being unable to communicate. Can you elaborate on this feeling?
12. Can you explain the initial feeling of understanding others but being unable to communicate with them?
13. When did you realize the 'true situation'? You mentioned experiencing depression upon understanding your reality and diagnosis. How long did it take to recover, and what aided your recovery? Could you expand on feeling like a burden and the sense of unfairness?
14. You now joke about the doctors giving you two months to live. How does it feel to joke about this now?
15. What was it like when you started regaining some movements?
16. You recalled a memory in the ICU involving your wife's sisters visiting. What do you remember about this? How did it make you feel? How does it feel knowing that other people continued living for many years post-diagnosis with the help of your book, and that doctors might have been aware of it?
17. What do you mean by 'more in-depth rehabilitation'? How do you envision the rehabilitation process by professionals? Which movements do you consider important, and do you believe you could have recovered with adequate rehabilitation?
18. You expressed a lack of psychological treatment during hospitalization. What aspects do you think needed addressing in psychological treatment? Did you seek a private psychologist after leaving the hospital? How did it help you? Do you find it easier to discuss your illness with a psychologist than with family and friends?
19. How did it feel when you tried to communicate with doctors, and they didn't respond? What did you mean by feeling like you reached a point where you couldn't improve upon discharge and returning home? Can you elaborate on the initial days/weeks at home? How did your parents' presence affect you?
20. During your nine months in the hospital, you were alone in your room. How do you think being in a shared room would have changed your experience?
21. Why do you mention currently 'surviving' in the residence? How do you feel about the residents believing you can hear them and their comments?
22. How did your family members' involvement make you feel during your hospitalization?
23. Can you elaborate on the process of learning to use the sheet and letter communication method? What was it like? You mentioned developing a common language with your wife—can you explain it and how you reached it? Describe the significance of your first sentence communicated via blinking. How did you experience this?
24. You mentioned that the decision to enter a residence was yours. Was it difficult to make this decision? How did you arrive at it? What differences do you notice between residences for the elderly and younger people? Why do you feel like you're 'surviving' in the residence? Do your needs get met? What are the most important needs, and which ones are fulfilled? Why do you think professionals and colleagues at the residence struggle to understand your condition?
25. How has the lack of medical care and rehabilitation post-hospitalization affected you? Do you believe this contributed to your movement limitations?
26. You mentioned giving up activities after the Captivity Syndrome diagnosis and said, 'it is when you see life normally'. Can you clarify this statement?
27. How do you feel about your wife attending all your friends' gatherings? What do you value most about the time spent with your friends?
28. You mentioned that, because of your condition, managers and rugby players hold a great affection for you. How does this affect you emotionally?
29. You mentioned developing a keen perception to discern if a family member or friend stays out of pity. Can you elaborate on this ability and how you've become attuned to these cues?
30. Could you clarify what you mean by "the situation you are experiencing in the residence?"
31. How do you feel about the minimal interaction and lack of patience from other residents in the residence when it comes to your communication efforts?
32. What emotions arise from the inability to engage with fellow residents, particularly in terms of their reluctance to assist with writing letters during conversations?
33. How has the ongoing COVID situation impacted life in the residence and your overall circumstances?
34. Regarding future plans, you mentioned having thoughts and surprises in life. Could you share some specific plans or an instance where life surprised you and how you felt about it?
35. Your wife mentioned a tape featuring your voice before the stroke. How does listening to it affect you emotionally?
36. Do you believe your relationship with your wife has improved since she is no longer your primary caregiver?
37. When dealing with architectural barriers, how do you cope with these challenges? Do you think people are generally aware of the difficulties faced by those with reduced mobility, and is society making efforts to address these obstacles?
38. Could you discuss other things in your life that you've come to value and their significance? Thank you for explaining the concept of "not telling your dreams."
39. Lastly, do you think there's an important topic I haven't addressed that you'd like to discuss?

**Questions from the semi-structured interview conducted with Participant 2:**

1. How did you become aware of your husband's stroke? What were your initial feelings upon discovering this?

2. Reflecting on the diagnosis of Locked-In Syndrome, what emotions and thoughts did you experience?

3. You previously mentioned withholding your husband's diagnosis initially. Could you elaborate on the reasoning behind that decision and how it made you feel?

4. Regarding your husband's nine-month hospitalization, what were your emotional shifts during this period? Could you describe your feelings from the beginning to the end of his hospital stay?

5. What specific treatments was your husband given during his hospitalization, and how would you evaluate their effectiveness?

6. How did you and your husband cope with his discharge from the hospital? What do you think his perspective was at that time?

7. When considering your husband's care, what were your expectations for his recovery during that period?

8. How did your social life change following his diagnosis?

9. As the primary caregiver, could you describe your experiences in that role?

10. What was your daily routine in the weeks leading up to your husband's move to the residence? Can you detail your activities on both workdays and weekends?

11. What prompted the decision to seek professional assistance and relocate your husband to a residence? How did you feel about this decision?

12. Reflecting on his current residence, how was the transition for both of you? How did you come to that decision, and who was involved in making it? How has life changed since his move to the residence?

13. How did you adapt to communicating with your husband through the letter boards and eye blinking method?

14. Were you and your husband enthusiasts of the same sports, particularly Rugby, before his stroke? Has this interest persisted?

15. Your husband previously discussed the architectural obstacles in your city. What are your perspectives on this issue?

16. How would you describe your current relationship dynamics? How do you assess your family and social life presently?

17. Your husband mentioned not making future plans anymore. How do you interpret this perspective, and does it resonate with you emotionally?

18. What are your personal expectations or plans for the future at this juncture?

#### COREQ CHECKLIST: “Locked-in syndrome: A qualitative study of a life story”

#### Consolidated criteria for reporting qualitative research (COREQ): a 32-item checklist for interviews and focus groups (http://www.equator-network.org/reporting-guidelines/coreq/).

#### Reference: Tong, A., Sainsbury, P. & Craig, J. (2007). Consolidated criteria for reporting qualitative research (COREQ): A 32-item checklist for interviews and focus groups. *International journal for quality in health care: journal of the International Society for Quality in Health Care*; 19(6):349-357. https://doi.org/10.1093/intqhc/mzm042.

**Consolidated criteria for reporting qualitative studies (COREQ): 32-item checklist**

| **NO. ITEM: GUIDE QUESTIONS/DESCRIPTION**  **GUIDE QUESTIONS/DESCRIPTION** | | **DONE** | **Location in the manuscript or supplementary material** |
| --- | --- | --- | --- |
| **DOMAIN 1: RESEARCH TEAM AND REFLEXIVITY** | | | |
| **Personal Characteristics** | | | |
| 1. Interviewer/facilitator: Which author/s conducted the interview or focus group? | ML-H and JP-C | **X** | **Supplementary material (COREQ CHECKLIST)** |
| 2. Credentials: What were the researcher's credentials? *E.g. PhD, MD* | ML-H (MD); CG-B, DP-C and JP-C (PhD) | **X** |  |
| 3. Occupation: What was their occupation at the time of the study? | ML-H (occupational therapist in neurological rehabilitation); CG-B and JP-C (occupational therapist and full university professors); DP-C (research nurse and full university professor) | **X** |  |
| 4. Gender: Was the researcher male or female? | ML-H and CG-B (females); DP-C and JP-C (males) | **X** |  |
| 5. Experience and training: What experience or training did the researcher have? | ML-H and CG-B (experience in the rehabilitation of people with neurological disorders); ML-H, CG-B, DP-C and JP-C (experience in qualitative research with people with neurological disorders) | **X** |  |
| **Relationship with participants** | | | |
| 6. Relationship established: Was a relationship established prior to study commencement? | The researchers had no prior relationship with the study participants | **X** | **Supplementary material (COREQ CHECKLIST)** |
| 7. Participant knowledge of the interviewer: What did the participants know about the researcher? e*.g. personal goals, reasons for doing the research* | Participants were aware of the researchers' motivations for conducting the study | **X** |  |
| 8. Interviewer characteristics: What characteristics were reported about the interviewer/facilitator? e.g. *Bias, assumptions, reasons and interests in the research topic* | Participants were previously informed about the researchers' interest in knowing and delving into the life history of a person with LIS, in its personal, emotional and functional impact, both from their perspective and from that of their wife, due to the absence of previous studies carried out in Spain | **X** |  |
| **DOMAIN 2: STUDY DESIGN** | | | |
| **Theoretical framework** | | | |
| 9. Methodological orientation and Theory: What methodological orientation was stated to underpin the study? *e.g. grounded theory, discourse analysis, ethnography, phenomenology, content analysis* | Qualitative life history study | **X** | **5** |
| **Participant selection** | | | |
| 10. Sampling: How were participants selected? *e.g. purposive, convenience, consecutive, snowball* | Purposive sampling | **X** | **5** |
| 11. Method of approach: How were participants approached? e*.g. face-to-face, telephone, mail, email* | Email and telephone | **X** | **Supplementary material (COREQ CHECKLIST)** |
| 12. Sample size: How many participants were in the study? | Two participants | **X** | **8** |
| 13. Non-participation: How many people refused to participate or dropped out? Reasons? | There were no dropouts or refusals to participate in the study | **X** | **Supplementary material (COREQ CHECKLIST)** |
| **Setting** | | | |
| 14. Setting of data collection: Where was the data collected? e*.g. home, clinic, workplace* | The interviews with the main participant were carried out by email and the interview with his wife was carried out in person at her home | **X** | **5** |
| 15. Presence of non-participants: Was anyone else present besides the participants and researchers? | The main participant responded to the interviews via email, being alone at the time of responding. During the interview with the wife of the main participant, only one of the researchers was present | **X** | **Supplementary material (COREQ CHECKLIST)** |
| 16. Description of sample: What are the important characteristics of the sample? *e.g. demographic data, date* | The description of sample is described in the Results section | **X** | **8-9** |
| **Data collection** | | | |
| 17. Interview guide: Were questions, prompts, guides provided by the authors? Was it pilot tested? | The question guide for the semi-structured interviews was carried out by reviewing the previous documents provided by the participants (books, radio interview and documentary film), reviewing the available scientific literature on LIS and with the clinical and research experience of the research team. No pilot tested was conducted | **X** | **Supplementary material (COREQ CHECKLIST)** |
| 18. Repeat interviews: Were repeat interviews carried out? If yes, how many? | Two semi-structured interviews were conducted with the main participant and one interview with his wife | **X** | **6-7** |
| 19. Audio/visual recording: Did the research use audio or visual recording to collect the data? | The interview with the wife was audio recorded | **X** | **7** |
| 20. Field notes: Were field notes made during and/or after the interview or focus group? | During the interviews, field notes were taken by the researcher | **X** | **6** |
| 21. Duration: What was the duration of the interviews or focus group? | The two interviews carried out with the main participant were by email. The recorded interview lasted 78 minutes. | **X** | **7** |
| 22. Data saturation: Was data saturation discussed? | Data saturation was not necessary due to the qualitative research method of the study (Qualitative life history study), in which a single participant and his wife were studied using the technique of crossed stories | **-** | **-** |
| 23. Transcripts returned: Were transcripts returned to participants for comment and/or correction? | Transcripts was returned to the participants, but they did not make any comments or corrections | **X** | **Supplementary material (COREQ CHECKLIST)** |
| **DOMAIN 3: ANALYSIS AND FINDINGS** | | | |
| **Data analysis** | | | |
| 24.Number of data coders: How many data coders coded the data? | Two researchers carried out the data analysis process | **X** | **Figure 1** |
| 25. Description of the coding tree: Did authors provide a description of the coding tree? | The description of the coding tree is shown in Figure 2 | **X** | **Figure 2** |
| 26. Derivation of themes: Were themes identified in advance or derived from the data? | An inductive thematic analysis was performed, therefore the themes were derived from the data | **X** | **7-8** |
| 27. Software: What software, if applicable, was used to manage the data? | No software was used for data analysis | **X** | **8** |
| 28. Participant checking: Did participants provide feedback on the findings? | Participants did not provide new feedback on the findings after data collection and analysis | **X** | **Supplementary material (COREQ CHECKLIST)** |
| **Reporting** | | | |
| 29. Quotations presented: Were participant quotations presented to illustrate the themes / findings? Was each quotation identified? e*.g. participant number* | All results were accompanied by quotes from the participants, identifying the participant number and the type of data collection source | **X** | **9-16** |
| 30. Data and findings consistent: Was there consistency between the data presented and the findings? | The consistency of the data and findings can be checked in the Results section | **X** | **9-16** |
| 31. Clarity of major themes: Were major themes clearly presented in the findings? | The themes and subthemes are described in the results section and shown in Figure 2 | **X** | **9-16, Figure 2** |
| 32. Clarity of minor themes: Is there a description of diverse cases or discussion of minor themes? | The themes and subthemes are described in the results section and shown in Figure 2 | **X** | **9-16, Figure 2** |
