# Supplementary material for: Locked‐in syndrome: A qualitative study of a life story
Source: Brain Behav. 2024 Aug 6;14(8):e3495. doi: 10.1002/brb3.3495 (PMC11302814; doi:10.1002/brb3.3495)
Supplement: Supplementary file 2 — Supplementary Material 2 [file BRB3-14-e3495-s002.docx]

**SUPPLEMENTARY MATERIAL 2.**

**Distribution of themes, subthemes and categories.**

| ***Themes*** | ***Subthemes*** | ***Categories*** |
| --- | --- | --- |
| ***Theme 1: Understanding and overcoming the new situation*** | Subtheme 1.1.: Being helped and accompanied by friends and family members. | Category 1.1.1.: Being accompanied in the hospital. |
|  |  | Category 1.1.2.: Parents helped me. |
|  |  | Category 1.1.3.: No one backed down. Feeling very loved. |
|  |  | Category 1.1.4.: Feeling valued and treated as before, without pity. |
|  | Subtheme 1.2.: Having a normal relationship, loving each other very much and making plans together. | Category 1.2.1.: Being friends and loving each other very much. |
|  |  | Category 1.2.2.: To have a normal relationship, even if it seems strange. |
|  |  | Category 1.2.3.: To have a strong social and family life. |
|  |  | Category 1.2.4.: To have plans to travel. |
|  |  | Category 1.2.5.: He wanted me to look for another man. |
|  |  | Category 1.2.6.: Newly married, we have plans. |
|  | Subtheme 1.3.: Understanding and coming to terms with the situation to cope with the disease. | Category 1.3.1.: To be aware of the real situation. |
|  |  | Category 1.3.2.: It was hard, a big blow, a huge depression. |
|  |  | Category 1.3.3.: Crying and suffering at the beginning, the world falls at your feet. |
|  |  | Category 1.3.4.: Understand that you have not been told the truth. |
|  |  | Category 1.3.5.: To be happy in the current situation, to accept oneself and to live. |
| ***Theme 2: The process of care and rehabilitation*** | Subtheme 2.1.: Recalling the first days after the stroke. | Category 2.1.1.: Do not remember anything. |
|  |  | Category 2.1.2.: It was horrible, but she was hard and strong. |
|  |  | Category 2.1.3.: To make the hospital your home. |
|  |  | Category 2.1.4.: Feeling bad and frustrated by the doctor's refusal. |
|  | Subtheme 2.2.: Valuing the rehabilitation process and the importance of different professionals. | Category 2.2.1.: Needed additional psychological care. |
|  |  | Category 2.2.2.: Importance of occupational therapy and physical therapy. |
|  |  | Category 2.2.3.: Treating you with affection and sharing emotions with nurses and assistants. |
|  |  | Category 2.2.4.: Importance of psychological treatment. |
|  |  | Category 2.2.5.: Lack of medical follow-up. |
|  |  | Category 2.2.6.: To be someone special, a rare case. |
|  | Subtheme 2.3.: Coming home. | Category 2.3.1.: Prepare the house for him and make him like in the hospital. |
|  |  | Category 2.3.2.: Drama, loss of privacy and truncated plans. |
|  |  | Category 2.3.3.: Feeling a burden, guilt and helplessness. |
|  | Subtheme 2.4.: As a caregiver. | Category 2.4.1.: Learning to take care of him. |
|  |  | Category 2.4.2.: Deterioration of physical and social life. |
|  | Subtheme 2.5.: Living in the residence. | Category 2.5.1.: Leaving for a nursing home because you feel a burden. |
|  |  | Category 2.5.2.: Get out more and feel less burdened. |
|  |  | Category 2.5.3.: Missing home. |
|  |  | Category 2.5.4.: Lack of empathy on the part of professionals. |
| ***Theme 3: Communication*** | Subtheme 3.1.: Inventing a system to communicate. | Category 3.1.1.: Communicating and knowing what you want is essential. |
|  |  | Category 3.1.2.: Yes and no, not real communication. |
|  |  | Category 3.1.3.: Inventing a flicker system as a communication method. |
|  |  | Category 3.1.4.: One of the best days of his life. |
|  |  | Category 3.1.5.: Need to have memory. |
|  | Subtheme 3.2.: Computer as a means of communication and contact with the world. | Category 3.2.1.: Importance of the computer to be able to communicate. |
|  |  | Category 3.2.2.: Programs to use the computer. |
|  |  | Category 3.2.3.: Computer to stay in touch with the world. |
| ***Theme 4: Writing as a way to help oneself and others*** | Subtheme 4.1.: Discovering the joy of writing to raise awareness of the disease. | Category 4.1.1.: A taste for writing. |
|  |  | Category 4.1.2.: Feeling useful and proud. |
|  | Subtheme 4.2.: Feeling useful and proud for helping yourself and others. | Category 4.2.1.: The book as a guide and as a help to accept the situation. |
|  |  | Category 4.2.2.: Feeling useful and proud for helping others. |
| ***Theme 5: Personal autonomy and social participation*** | Subtheme 5.1.: Dependency and supports for basic activities of daily living. | Category 5.1.1.: Impairment of the basic activities of daily living. |
|  |  | Category 5.1.2.: To be dependent. |
|  |  | Category 5.1.3.: Support products for a new life. |
|  | Subtheme 5.2.: Mobility in the community. | Category 5.2.1.: Eliminate and avoid architectural barriers. |
|  |  | Category 5.2.2.: It is complicated, there are many things in bad condition. |
|  |  | Category 5.2.3.: An uncomfortable chair that I couldn't stand. |
|  |  | Category 5.2.4.: Have the right and capacity to vote. |
|  | Subtheme 5.3.: Difficulties in emotional management and social interaction. | Category 5.3.1.: Lack of control of emotions. |
|  |  | Category 5.3.2.: Hurtful words and compassion. |
